# Preliminary Outcomes of Cervical Cerclage for Shortened Cervix with Decidual Polyp

**DOI:** 10.3390/healthcare10071312

**Published:** 2022-07-14

**Authors:** Takuya Misugi, Kohei Kitada, Megumi Fudaba, Sayaka Tanaka, Yasushi Kurihara, Mie Tahara, Akihiro Hamuro, Akemi Nakano, Masayasu Koyama, Daisuke Tachibana

**Affiliations:** 1 Department of Obstetrics and Gynecology, Osaka Metropolitan University Graduate School of Medicine, 1-4-3 Asahimachi Abeno-ku, Osaka 545-8585, Japan; g21621t@omu.ac.jp (K.K.); mecch1n@outlook.jp (M.F.); v21555o@omu.ac.jp (Y.K.); mtahara@omu.ac.jp (M.T.); hamuroa@omu.ac.jp (A.H.); akeake@omu.ac.jp (A.N.); k21322v@omu.ac.jp (M.K.); dtachibana@omu.ac.jp (D.T.); 2 Department of Pathology, Osaka Metropolitan University Graduate School of Medicine, 1-4-3 Asahimachi Abeno-ku, Osaka 545-8585, Japan; sayka.design@omu.ac.jp

**Keywords:** cervical cerclage, decidual polyp, endocervical polyp, preterm birth

## Abstract

The aim of this study was to elucidate the nature of decidual polyp (DP) and to compare DP outcomes treated with cervical cerclage for a shortened cervix with the outcomes of cases treated with cervical cerclage without DP. The medical records of pregnant women who were complicated with cervical polyps were retrospectively reviewed. Cervical cerclage was considered for those cases with a shortened cervical length of under 25 mm and before 25 gestational weeks. We also reviewed pregnant women who had no cervical polyps, and who underwent cervical cerclage during the same study period, and defined them as the control group. A total of 56 pregnant women with cervical polyps were identified. All of the polyps in the 14 cases that had undergone cervical cerclage migrated into the cervical canal. Of the thirty seven cases with cervical polyps that did not necessitate cervical cerclage, eight women delivered preterm and six of these cases were diagnosed as DP. In all of the women studied, polyp migration was observed in 68.6 %. Cervical cerclage was performed significantly earlier in the DP group than in the control group of 46 cases (*p* < 0.001; 18.4 weeks vs. 21.4 weeks, respectively). Cervical cerclage is effective in DP cases with a shortened cervical length and polypectomy should not be performed during pregnancy because of the risk of miscarriage.

## 1. Introduction

Uterine cervical polyps are a common asymptomatic condition in the gynecological practice and sometimes present with vaginal bleeding and increased discharge [1,2,3]. Most of the polyps are benign and resection for pathological examination is commonly performed in non-pregnant women, because the polypectomy itself is painless and does not take much time [2,3]. It is hypothesized that these polyps are associated with chronic inflammation, reaction to foreign bodies, a localized congestion of cervical vasculature and the abnormal local response to estrogen stimulation. However, the exact causes of uterine cervical polyps remain unclear [4,5]. Several authors have recently reported that, during pregnancy, cervical polyps are highly associated with adverse perinatal outcomes, such as spontaneous abortion and preterm delivery, and that a polypectomy increases these risks, especially in cases where the polyps are composed of decidua (decidual polyp: DP) [6,7,8,9]. However, insight into the management and complications of polyps protruding from the cervical canal during pregnancy is limited.

The aim of this study was to elucidate the nature of DP and to compare DP outcomes treated with cervical cerclage for a shortened cervix with the outcomes of cases treated with cervical cerclage without DP in order to validate the efficacy of the treatment.

## 2. Materials and Methods

This retrospective observational study was approved by the institutional review board (Approved Number: 2020-50, June 2020), and all patients provided their informed written consent. The medical records of pregnant women who were complicated with cervical polyps and delivered in Osaka Metropolitan University Hospital between September 2016 and April 2022 were retrospectively reviewed. Multiple pregnancies were excluded because there were various confounding factors in multiple pregnancies and preterm births.

**Figure 1:**
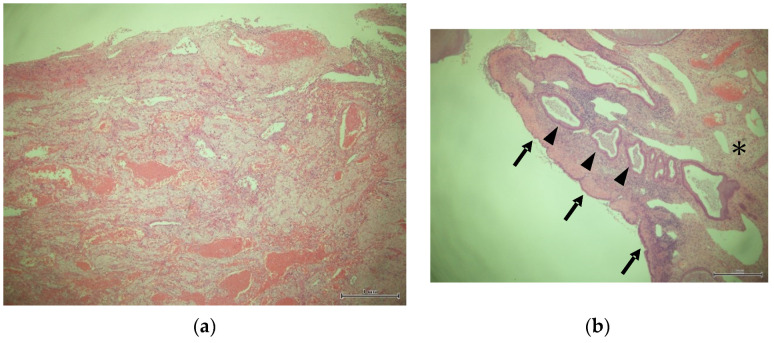
Decidual polyp showing abundant pink cytoplasm without epithelium and/or glandular structures (**a**) and endocervical polyp containing decidual change in stromal cells (* in (**b**)). Arrows indicate endocervical epithelium covering a fibrovascular core and arrowheads indicate glandular structures within the endocervical polyp.

At our institution, cervical polyps recognized during pregnancy were closely monitored in cases where the cytology of the polyps did not show any malignant potential. Cervical cerclage via the McDonald technique was considered for those cases with a shortened cervical length of under 25 mm and before 25 gestational weeks. Vaginal swabs were performed as a screening test for bacterial vaginitis if signs of threatened preterm labor, such as shortened cervical canal length, were recognized. Cervical cerclage was avoided if the patients complained of any uterine activity and/or infectious signs. A specimen taken from the polyp during the cerclage procedure was pathologically examined. Operators did not intentionally push the polyps into the cervical canal, and polyps were sometimes left protruding even after the cerclage. In cases where the polyps were thought to disappear with the progression of gestation, as identified via vaginal inspection, the uterine cervical canal was carefully observed by a trans-vaginal ultrasound to detect the migration of the polyp into the canal. If the pregnancy extended and the polyps were visible by vaginal inspection until 36 to 37 weeks, a polypectomy was performed. DP was defined as polyps with cells showing abundant pink cytoplasm without epithelium and/or glandular structures (Figure 1a) [6]. If the polyp was covered with glandular epithelium on its surface and/or contained glandular structures, the polyp was diagnosed as an endocervical polyp, even if it contained any decidua-like changes in the stromal cells (Figure 1b). For the comparison of cerclage efficacy for DP cases, we reviewed the pregnant women who had no cervical polyps, and who underwent cervical cerclage via the McDonald technique during the same study period and defined them as the control group.

Continuous variables were expressed as median (range), and categorical variables were expressed as numbers (%). Differences between the DP group and the control group were studied using Fisher’s exact test for categorical variables and the Mann–Whitney U test for continuous variables. A *p* value of <0.05 was considered to be statistically significant. An analysis was carried out with the SPSS statistics version 20 (SPSS Inc., Chicago, IL, USA).

## 3. Results

**Figure 2:**
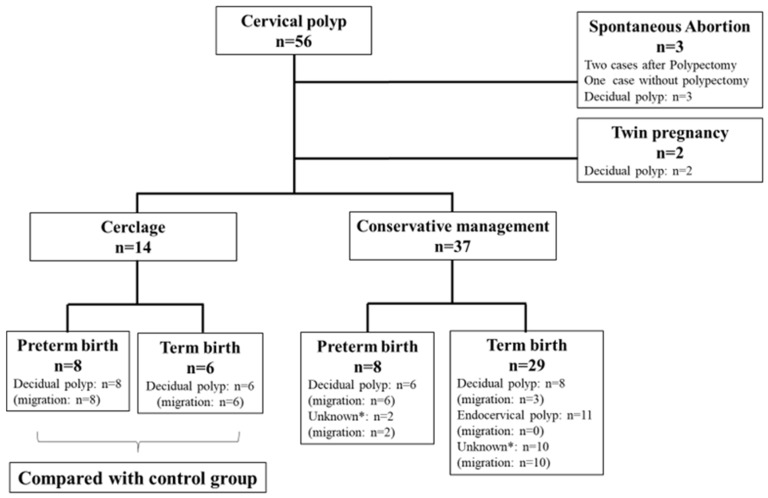
Patient flow diagram. * Unknown: cases without pathological confirmation because of migration and/or severe degeneration.

During the study period, a total of 56 pregnant women with cervical polyps were identified, and their clinical features and pathological outcomes are shown in Figure 2. Three cases with DP resulted in a spontaneous abortion between 13 and 17 gestational weeks, and two of these cases underwent polypectomy at referral clinics. All of the polyps in the 14 cases that underwent cervical cerclage migrated into the cervical canal before and/or after operation, and all of these polyps were diagnosed as decidual; eight of the women delivered preterm. Of the thirty seven cases with cervical polyps that did not necessitate cervical cerclage, eight women delivered preterm and six of these cases were diagnosed as DP. In all of the women studied, polyp migration was observed in 35 cases (68.6%) and a pathological diagnosis was not possible in 12 cases because of the difficulty of recognizing the polyps and the severity of polyp degeneration. Within the 16 cases where the polyps were left protruding (Figure 2), 11 of these were found to be endocervical polyps.

**Figure 3:**
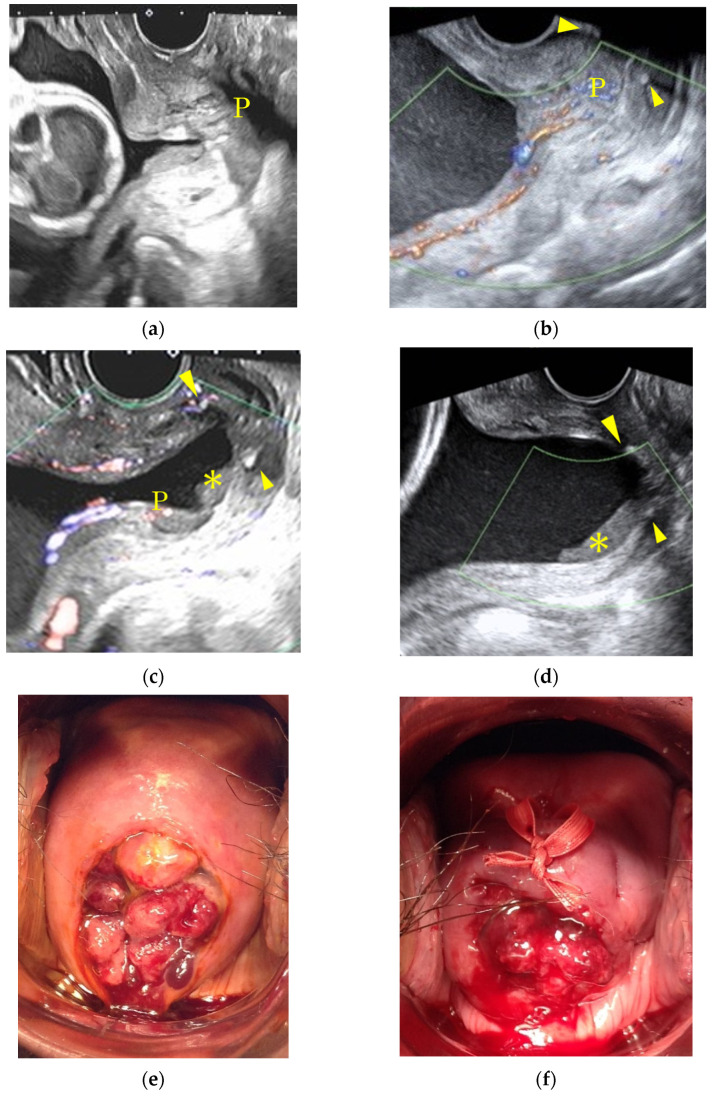

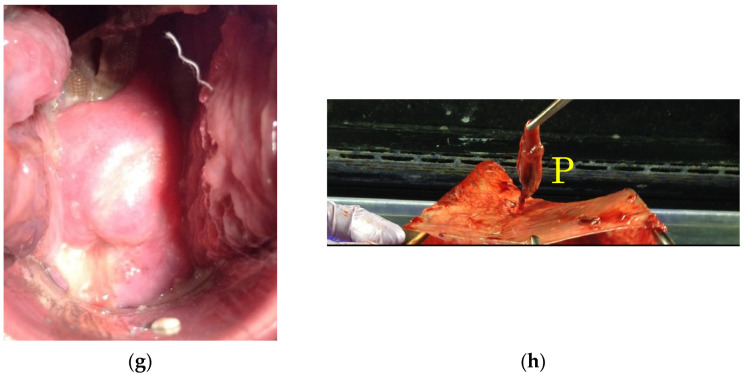
Trans-vaginal ultrasound images show decidual polyps protruding from the intra-uterine cavity and a shortened cervix, which necessitated a cervical cerclage, observed at 17 weeks of gestation. (**a**), the polyps migrated inside the area of the cervical canal at 22 weeks of gestation; (**b**), and further migrated upward at 26 weeks of gestation (**c**). They could not be recognized anymore by a trans-vaginal ultrasound at 29 weeks of gestation (**d**). Speculum examination during the operation showed multiple polyps (**e**) and their protrusion even after the cerclage procedure at 17 weeks of gestation (**f**); however, polyps were not visible at 22 weeks of gestation (**g**). A decidual polyp was found attached to the amniotic membranes at the placental macroscopic inspection (h). Arrow head: string of cervical cerclage, P: decidual polyp, *: sludge.

Table 1 shows the comparisons of maternal characteristics and cerclage outcomes between the study groups, and Table 2 shows the comparisons of neonatal outcomes between them. Cervical cerclage was performed significantly earlier in the DP group than in the control group (*p* < 0.001; 18.4 weeks vs. 21.4 weeks, respectively), and the duration from cerclage to delivery was significantly longer in the DP group than in the control group (*p* = 0.004; 19.0 weeks vs. 14.9 weeks, respectively). However, there was no significant difference between the two groups regarding the perinatal outcomes. A typical case which showed the migration of polyps after cervical cerclage is presented in Figure 3.

Table 3 shows the details of patients who underwent cervical cerclage with DP. Two pregnant women who were complicated with a cervical polyp had a previous history of spontaneous abortion and preterm delivery. None of the cases complained of uterine activity nor showed any infectious signs. Furthermore, the diagnosis of cervical polyps was made at 9.7 gestational weeks at median, and migration was observed in all cases as early as 23 gestational weeks at median.

## 4. Discussion

The results of this study show that the rate of pathologically confirmed DP was as high as 54.9% (28/51). Moreover, 27.5% of DP cases (14/51) necessitated cervical cerclage at as early as 18.5 gestational weeks, and cervical cerclage for DP cases was similarly effective as that for non-DP cases in the control group, with both leading to satisfactory perinatal outcomes. Furthermore, migration of the polyp into the cervical canal was observed in 35 cases (68.6%) among all of the pregnant women in the study with cervical polyps.

Tokunaka et al. investigated which types of cervical polyps (removed during the first and second trimesters) were associated with the risk of spontaneous abortion and preterm delivery. They found that the resection of DPs was a significant risk factor (adjusted odds ratio: 13.86, 95% confidence interval: 2.91–105.50) [6]. More recently, Fukuta et al. reported that the clinical features of risks for spontaneous abortion and preterm delivery after first trimester polypectomy were polyp size (width > 12 mm) and the pathology of the DP [8]. These two reports suggested that the conservative management of cervical polyps might be safer in cases where a malignant tumor is not suspected. In addition to these reports, our study showed that when DPs were not removed, migration was frequently observed with the progression of gestation. In other words, migration may be an indirect sign of its origin from the uterine cavity.

Obstetricians are faced with a dilemma in that performing a polypectomy for pregnant women, which is a requisite for differentiating malignancy, might be associated with spontaneous abortion and preterm delivery. Furthermore, the diagnosis of DP carries some difficulty because stromal decidual change may occur even in endocervical polyps [1]. However, as shown in Figure 1, we were able to differentiate endocervical polyps from DPs with the finding of endocervical epithelium covering a fibrovascular core and glandular structures within the polyp itself. Furthermore, a trans-vaginal ultrasound may provide additional information such as an image of the roots of cervical polyps connecting to the decidua, a strong indication that the polyp is DP [9,10]. After the confirmation of benign cytology derived from its surface, the cervical polyp can then be managed conservatively.

The meta-analysis revealed that intravaginal progesterone therapy in cases of shortened cervical length is effective in preventing preterm delivery [11], but its efficacy in preventing preterm delivery in Asians has not been confirmed. There have been no large-scale studies of progesterone therapy conducted in Japan, and vaginal tablets have not been approved in Japan for the treatment of threatened preterm labor. Therefore, we do not treat threatened preterm labor with shortened cervical length with progesterone, but with cervical cerclage.

The limitation of this study is that the number of the patients included was relatively small to establish solid evidence for the management of DP. However, our study was the first to reveal that cervical cerclage may be effective in prolonging the duration of pregnancy and thus could achieve better perinatal outcomes.

## 5. Conclusions

In conclusion, we recommend the conservative management of cervical polyps during pregnancy and after the confirmation of benign cytology. Furthermore, if the patient shows a cervical shortening in the previable period far from term, cervical cerclage without a polypectomy should be considered.

## Figures and Tables

**Table 1:** Comparisons of maternal characteristics and cerclage outcomes between the study groups.

	DP Group (*n* = 14)	Control Group (*n* = 46)	*p* Value
Age (year)	36.0 (27–42)	35.0 (20–49)	0.982 ^a^
Body mass index (kg/m^2^)	22.8 (17.8–36.9)	20.9 (16.2–34.2)	0.393 ^a^
Gravida	2 (1–5)	2 (1–6)	0.42 ^a^
Parity	1 (0–3)	1 (0–4)	0.575 ^a^
ART (n)	2 (14.3%)	10 (16.9 %)	1 ^b^
Cervical length at cerclage	15 (9–24)	17.3 (4.0–24.0)	0.624 ^a^
WBC at cerclage (/μL)	8500 (7300–13,300)	8700 (5200–16,000)	0.259 ^a^
CRP at cerclage (mg/dL)	0.14 (0.02–0.75)	0.13 (0.01–1.99)	0.988 ^a^
Gestational week at cerclage (week)	18.4 (13.0–22.9)	21.4 (17.0–24.9)	<0.001 ^a^
Duration of tocolysis (day)	8 (4–119)	8 (1–92)	0.375 ^a^
Duration from cerclage to delivery (week)	19.0 (8.7–23.4)	14.9 (2.7–22.0)	0.004 ^a^
Gestational week at delivery (week)	36.9 (27.6–41.9)	37.1 (24.3–41.1)	0.982 ^a^

a: Mann–Whitney U test; b: Fisher’s exact test; ART: assisted reproductive technology; CRP: C-reactive protein.

**Table 2:** Comparisons of neonatal outcomes between the study groups.

	DP Group (*n* = 14)	Control Group (*n* = 46)	*p* Value
Birthweight (g)	2530 (884–3425)	2815 (600–3780)	0.197 ^a^
Apgar score at 1 min	8 (1–9)	8 (1–9)	0.088 ^a^
Apgar score at 5 min	9 (4–10)	9 (1–10)	0.64 ^a^
pH of Umbilical artery	7.29 (7.18–7.36)	7.29 (7.09–7.41)	0.812 ^a^

a: Mann–Whitney U test.

**Table 3:** Details of patients with decidual polyps treated with cervical cerclage.

Patient	Age	Parity	Diagnosis (Week)	Migration (Week)	Cerclage (Week)	CL at Cerclage (mm)	Delivery (Week)	CAM
1	37	0	7.3	21.4	21.4	24	35.1	Non
2	34	0	7.3	19.0	19.0	13	36.0	Stage1
3	30	2	7.4	20.1	20.1	20	36.9	N/A
4	40	1	7.7	17.6	17.6	16	39.1	Stage2
5 ^a^	36	0	8.0	15.6	15.6	14	37.9	Non
6	28	0	8.6	34.4	14.9	22	38.3	Stage2
7 ^b^	42	3	9.4	13.0	13.0	22	35.0	Non
8	37	1	10.1	14.1	18.1	0 ^c^	36.7	Stage2
9	34	0	10.3	14.6	18.7	10	37.7	Stage3
10	37	1	10.6	20.7	20.7	24	39.9	N/A
11	37	1	12.0	32.9	18.4	18	41.9	Stage2
12 ^b^	27	0	17.9	21.1	18.0	9.0	33.0	Stage2
13	36	0	19.1	19.1	19.1	10	28.0	Non
14	28	0	22.7	25.7	22.9	14	27.6	Non
Median	36	1	9.8	19.6	18.6	16	36.8	

DP: decidual polyp; N/A: not available; CAM: pathologically diagnosed chorioamnionitis. a: conceived by assisted reproductive technology; b: the patients who have a previous history of spontaneous abortion or preterm delivery with cervical polyps; c: the amniotic membrane was observed at the level of external cervical ostium.

## Data Availability

Not applicable.
